# Development and validation of an assessment tool for public perceptions toward drive-thru community pharmacy services in Malaysia during COVID-19

**DOI:** 10.3389/fpubh.2023.1144466

**Published:** 2023-08-03

**Authors:** Bayan Faisal Ababneh, Siew Chin Ong, Louai Alsaloumi, Rabia Hussain

**Affiliations:** ^1^Discipline of Social and Administrative Pharmacy, School of Pharmaceutical Sciences, Universiti Sains Malaysia, Penang, Malaysia; ^2^Discipline of Clinical Pharmacy, Faculty of Pharmacy, Near East University, Nicosia, Northern Cyprus, Türkiye

**Keywords:** confirmatory factor analysis, exploratory factor analysis, drive-thru pharmacy, validity, reliability

## Abstract

**Introduction:**

Community pharmacists' roles have expanded and undergone a significant transition over the last few years. Consequently, new and different pharmacy services, such as drive-thru pharmacy services, have emerged. Drive-thru pharmacy services began three decades ago and continued even during outbreaks such as the COVID-19 pandemic. Patients' perceptions are essential to the successful implementation and satisfaction with any new service. This study examines the reliability and validity of the assessment tool of public perceptions toward drive-thru community pharmacy services in Malaysia during COVID-19.

**Methods:**

A cross-sectional study was conducted. The developed tool consists of 28 items to evaluate believed advantages toward drive-thru community pharmacy services, believed disadvantages toward drive-thru community pharmacy services, differences between drive-thru community pharmacy services and instore drug refill services, perceptions toward drive-thru community pharmacy services and feelings regarding how the introduction of drive-thru pharmacy services may affect the image of community pharmacists. Exploratory factor analysis (EFA) was performed to identify the factors of the developed tool, and confirmatory factor analysis (CFA) evaluated the model fitness.

**Results:**

The EFA identified five elements and 25 items for the tool, and through CFA results, the observed model of the 25 items structure of the tool was verified as an excellent fit for the data [χ^2^ (265, *N* = 565) = 819.586, *p* < 0.001, IFI = 0.931, CFI = 0.93, RMSEA = 0.064]. The results of the CFA indicated a good model fit between the observed model and the proposed model. The internal reliability of the entire tool and each factor was very satisfactory as Cronbach's Alpha for the whole structured tool was 0.843 and for each factor was as follows, first factor (believed advantages) = 0.909, second factor (believed disadvantages) = 0.921, third factor (differences between drive-thru and instore refill) = 0.647, fourth factor (perceptions) = 0.926, and fifth factor (feelings) = 0.681.

**Conclusion:**

The developed and validated tool would be valuable for assessing the public's perceptions of the drive-thru community pharmacy service during COVID-19 and future pandemics.

## Introduction

Community pharmacists' roles have expanded and undergone a significant transition over the last few years. The roles were previously limited to mainly compounding and dispensing medications (1). However, the new transition in pharmacy practices has shifted the focus to patient care (1) to promote the quality of healthcare services and improve the control of illnesses (2).

Extended pharmacy services (EPS) are services provided at pharmacies other than traditional services (for example, dispensing prescribed or over-the-counter medications and providing counseling or instructions about dispensed medications) (3, 4). EPS include identifying medication-related problems by conducting comprehensive medication reviews, monitoring diseases such as hypertension by measuring blood pressure levels or diabetes mellitus by measuring blood glucose levels, pain management and wound care, diet and healthy lifestyle services, and contacting the primary healthcare team (3–5). In addition to EPS, there are newly added services at pharmacies, such as drive-thru pharmacy services (6).

Many countries have had drive-thru pharmacy services in a community setting for ~30 years (6). Drive-thru pharmacy services began in the United States in the 1990s to improve the accessibility of healthcare services to older adults (6). Later, the service was adopted by different countries such as Australia (7), Croatia (8), Jordan (9), Malaysia (10, 11), Taiwan (12), and the United Kingdom (13). It was introduced to reduce waiting times, solve parking problems, and improve the accessibility of healthcare services to working parents and older adults (14–16). The Pharmaceutical Services Division of the Ministry of Health of Malaysia initiated the first drive-thru pharmacy services in Malaysia in 2003 as part of the pharmacy value-added service (VAS) (17). During the COVID-19 pandemic, it was introduced by community pharmacies in Malaysia (18). The head of the Malaysian Pharmacy Association stated that the drive-thru pharmacy service is the best way to access health services during the COVID-19 pandemic to minimize infection rates (18). The first community pharmacy to initiate the service was Superbig Kubang Kerian Pharmacy in 2022 (18). Additionally, like Malaysia, some countries, such as Qatar and the United Arab Emirates (UAE), initiated drive-thru pharmacies for the first time to ensure the safety of pharmacists and consumers during COVID-19 (15, 16).

The assessment of pharmacy services can be judged by regulatory agencies, consumers, and service providers (19). However, to assess the quality of healthcare services, patient satisfaction is considered a valuable indicator, as it affects clinical outcomes coupled with efficient, timely, and patient-centered healthcare service delivery (20). Therefore, to evaluate new services, it is important to consider patients' perceptions (21, 22). Previous studies have demonstrated positive patients' perceptions of drive-thru pharmacy services in hospitals or communities (9–11, 23, 24). The Queen Elizabeth Hospital (QEH), Kota Kinabalu, offered a drive-thru pharmacy service in 2015. Liew et al. propounded that patients who used the drive-thru pharmacy service at QEH were satisfied with it (11). Another study in Malaysia was conducted in Hospital Raja Perempuan Zainab II (HPRZ II), in which patients were aware of the presence of a drive-thru pharmacy service and the importance of its public use (10). A cross-sectional study was conducted in Saudi Arabia to evaluate the need for drive-thru pharmacy services during COVID-19; the result suggested a crucial need to support the community pharmacy with drive-thru pharmacy services. However, this result is limited, because the survey was only conducted in Saudi Arabia (24).

Positive feedback toward using drive-thru community pharmacy services was reported by Jordanian pharmacy customers, especially busy customers who were male, married, and had children, as they confirmed that such services are fast and time-saving (23). However, drive-thru pharmacies may reduce interactions between the pharmacist and patient, significantly affecting the counseling process (25). This evidence was further supported by assessing awareness, perception, and barriers among pharmacists in Jordan (9). On the other hand, drive-thru pharmacy services could provide convenient dispensing of medications and solve the limited parking slots problem, thereby improving patient satisfaction (9).

Due to the availability of limited studies that have evaluated the public's perceptions toward drive-thru pharmacy services within the last 10 years (10, 11, 23, 24), more research is needed to assess the public's experiences toward drive-thru pharmacy services (15). Moreover, there is a lack of attention to drive-thru services in the community pharmacy setting, particularly during the COVID-19 period in Malaysia. The lack of awareness is caused by previous studies in Malaysia focusing solely on the drive-thru pharmacy in government hospitals and before COVID-19 (10, 11).

Additionally, the tools used to assess the public's perceptions of drive-thru pharmacy services in the hospital and community settings before COVID-19 were validated and consisted of demographics, the public's satisfaction level of drive-thru pharmacy services, experience evaluation, and perceived advantages and disadvantages of drive-thru pharmacy services (10, 11, 23). Thus, it is crucial to develop a tool to assess perceptions of drive-thru community pharmacy services during COVID-19 from the public's perspective. Therefore, this study aims to develop this tool to evaluate the public's perceptions in Malaysia and to validate this measure using the modern test theory.

## Methodology

### Study design and data collection

A cross-sectional study was conducted. A self-administered tool using an online Google form was utilized for data collection. It was then distributed to the participants by research assistants [undergraduate pharmacy students at Universiti Sains Malaysia (USM)] via social media platforms such as WhatsApp, Instagram, and Telegram. To ensure the completeness of the participants' responses and that all questions were answered with no missing entries, the form was preoptimized through a Google form option, where all items must be filled out before submitting the participant's response. The time to complete the form was ~5–7 min.

### Participants and setting

The study was conducted in Malaysia between 19 May and 22 June 2022. All citizens who currently reside in Malaysia were invited to participate in the study. The inclusion criteria comprised participants aged 18 years and above, who had access to the Internet via a computer or smartphone to answer the survey through online platforms, and who could read and understand English. All participants voluntarily participated in this study, and no incentives were offered for participation.

### Sampling and sample size

A non-probability convenience sampling method was used for data collection. The sample size calculation was determined using the following formula (26), which revealed that the sample size needed to be at least 363 participants: *n* = *z* 2 × ρ* ˆ* (1−ρ* ˆ*) ÷ ε2, where *z* is the z score, ε is the margin of error, *n* is the population size, and ρ* ˆ* is the population proportion. *z* for a confidence level of 95% was 1.96. The margin of error was 5%. We assumed a population proportion of 0.6, as one study in Malaysia revealed 60% awareness regarding the drive-thru service (10), and an unlimited population size. In case of missing data, a higher sample size was recruited (*n* = 565).

### Tool development and validation

The tool was developed by the research team based on the recommended process of tool development by Davis (27). The process was as follows: (1) the identification of the tool concept by a thorough literature review (9–11, 23, 24); (2) determining the formatting, writing, scoring, and comprehensibility; (3) performing validity tests by an expert panel and conducting factor analyses (CFA and EFA); and (4) performing the reliability test. The research team consulted some community pharmacists working in Malaysia to add some questions to the tool, and all their suggestions were incorporated into the developed tool. The tool was designed, validated, and presented in English. Furthermore, experienced academicians at USM were invited to review the questionnaire's content before distribution to the targeted participants and to ensure the developed tool's comprehensibility and face and content validity (28). Any amendments were made based on the feedback and suggestions received.

The developed tool consisted of three main sections. This included the participants' demographic information, attitudes toward drive-thru community pharmacy services, and perceptions toward drive-thru community pharmacy services. Items for the first and second sections, which were socio-demographic information and attitudes of the general public toward drive-thru community pharmacy services, were designed as open-ended and multiple-choice questions. The third section consisted of 28 items and discussed five factors, which were as follows: (1) the believed advantages of drive-thru community pharmacy services, (2) the believed disadvantages of drive-thru community pharmacy services, (3) the differences between drive-thru community pharmacy services and in-store drug refill services, (4) the perceptions toward drive-thru community pharmacy services, and (5) the feelings regarding how the introduction of drive-thru community pharmacy services may affect the image of community pharmacists. This section was designed through a 5-point Likert Scale to indicate the degree of agreement or disagreement of the participants with each statement in this section. The responses ranged from 1 to 5 (5 = strongly agree, 4 = agree, 3 = neutral, 2 = disagree, and 1 = strongly disagree), and a reverse coding was considered for the negative statements and the disadvantages (1 = strongly agree, 2 = agree, 3 = neutral, 4 = disagree, and 5 = strongly disagree).

To identify and resolve the potential problems and deficiencies in the developed tool, a pilot test was conducted among 36 participants to test the developed tool before distribution to the targeted participants (29). The participants in the pilot test were excluded from the final analysis.

To ensure the reliability of the developed tool, internal consistency was performed, which measured how closely related the items were for each domain. It was calculated using Cronbach's alpha coefficient (28), with values of 0.70 and above indicating good internal consistency (30). The results of reliability are presented in the results section.

### Data analysis

Exploratory factor analysis (EFA) was performed using the IBM Statistical Package for Social Science, version 28 (SPSS Inc., Chicago, IL, USA). Furthermore, confirmatory factor analysis (CFA) was conducted using Analysis of Moment Structures (AMOS) version 28.

EFA is a generating theory in which possible relationships between variables in a developed tool can be identified. In contrast, CFA is a testing theory in which the relationships between variables in a developed tool can be tested (31).

First, to assess the appropriateness of the data for factor analysis, the Kaiser–Meyer–Olkin measure of sampling adequacy (>0.5) and Bartlett's test of sphericity (< 0.05) were performed (32). The maintained factors in the model were identified through the principal component analysis (28). In total, 28 items for a 565-sample size were tested, and the considered factors had an eigenvalue of >1 (33). Five factors were considered, and EFA with oblimin rotation was performed to evaluate the theoretical structure of the tool. Subsequently, CFA was performed to validate previously considered factors using maximum likelihood estimation (34). The models' goodness of fit was identified by using several statistics such as the overall chi-square (χ^2^), root mean square error of approximation (RMSEA), comparative fit index (CFI), and incremental fit index (IFI) (34–36). For CFI and IFI, the values ranged from 0 to 1, and being closer to 1 meant a higher relationship between variance and covariance (37, 38). The excellent model fit was indicated by the RMESA value being equal to or < 0.06 (39, 40), and the degree of model fit was indicated by reporting the IFI (35).

### Ethical consideration

Study approval was obtained from the Human Research Ethics Committee of USM (Reference code: USM/JEPeM/21110755). Moreover, the participants who agreed to participate in the study signed the consent form electronically before proceeding to the first section of the tool. The participants could have exited the survey anytime if they refused to answer the questions.

## Results

### Response rate

Of note, 800 surveys were distributed to the Malaysian public. A total of 565 (70.6% response rate) members of the general public completed the survey.

### Exploratory factor analysis for validity

**Figure 1:**
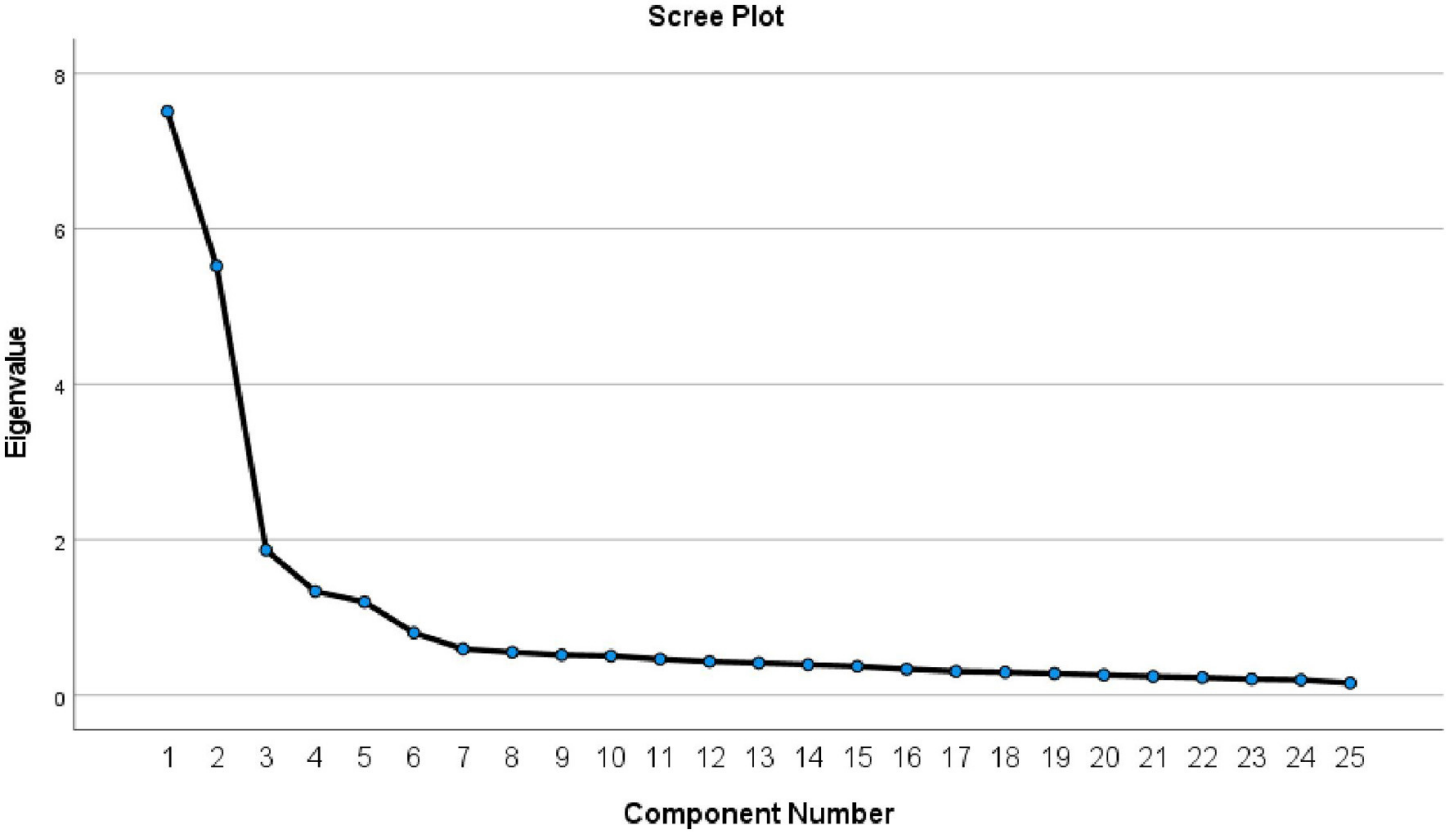
Scree plot for the tool.

Before conducting the EFA, the appropriateness of the sample size for performing EFA was checked through the Kaiser–Meyer–Olkin (KMO) measure. The result of KMO verified the sampling adequacy for the analysis, KMO = 0.917. Bartlett's test of sphericity, χ2_(378)_ = 8,838.195, *p* < 0.000, indicated that the correlations between the items were strong, and the data were suitable for conducting the EFA (32, 41).To identify factors that should be kept, the principal component analysis was performed on 28 items with the oblimin rotation of EFA (33, 42), as shown in Figure 1.

An initial analysis was run to obtain eigenvalues for each factor in the data, which showed that five elements had eigenvalues over 1.

The findings of the factor loadings by conducting the EFA are presented in Appendix 1. The first factor consisted of six items that represented the believed advantages of drive-thru community pharmacy services. The second factor consisted of seven items representing the believed disadvantages toward drive-thru community pharmacy services. The third factor consisted of five items representing differences between drive-thru community pharmacy services and in-store drug refill services. The fourth factor comprised five items representing perceptions of drive-thru community pharmacy services. The fifth factor consisted of two items representing feelings regarding how the introduction of drive-thru community pharmacy services may affect the image of community pharmacists. The final five-factor structure was composed of 25 items after deleting three items cross-loaded on multiple factors and a loading factor < 0.5.

The first deleted item, < 0.5 (38, 43), was “*Community pharmacists will appear more concerned with making money than with the health of their patients”*, because it had a factor loading of 0.454^*^ on the fifth factor and a cross-loading of 0.170 on the first factor. The second deleted item was “*Drive-thru service provides accessibility and convenience to customers more than the in-store service, especially during COVID-19 time”*, because it had a factor loading of 0.341^*^ on the first factor and a cross-loading of 0.211 on the third factor. The third deleted item was “*The prescription might be filled more quickly in drive-thru compared to in-store refill”*, because it had a factor loading of 0.343^*^ on the fourth factor and a cross-loading of 0.232 on the first factor.

Finally, this 25-item structure explained 69.74% of the variance in the pattern of relationships among the items. The percentages explained by each factor were 30.03% (factor one, believed advantages), 22.08% (factor two, believed disadvantages), 7.47% (factor three, differences between drive-thru community pharmacy services and in-store drug refill services), 5.34% (factor four, perceptions), and 4.79% (factor five, feelings). The final tool is available in Appendix 2.

### Item analysis for reliability

Table 1 presents the conducted reliability test analysis for each factor and the structured tool. The satisfactory internal consistency ranged from 0.7 to 0.9 (30). All five factors in this study had high-reliability values. Cronbach's alpha of the first factor (believed advantages), second factor (believed disadvantages), third factor (differences), fourth factor (perceptions), and fifth factor (feelings) was 0.909, 0.921, 0.647, 0.926, and 0.681, respectively. Moreover, Cronbach's alpha of the entire structured tool was 0.843.

**Table 1:** Cronbach's alpha for each factor in the tool.

**Factor**	**cronbach's Alpha**	**Cronbach's alpha, based on standardized items**	**Number of items**
First factor	0.909	0.911	6
Second factor	0.921	0.921	7
Third factor	0.793	0.792	5
Fourth factor	0.926	0.928	5
Fifth factor	0.681	0.688	2
Total tool	0.844	0.851	25

### Confirmatory factor analysis for predictive validity

**Figure 2:**
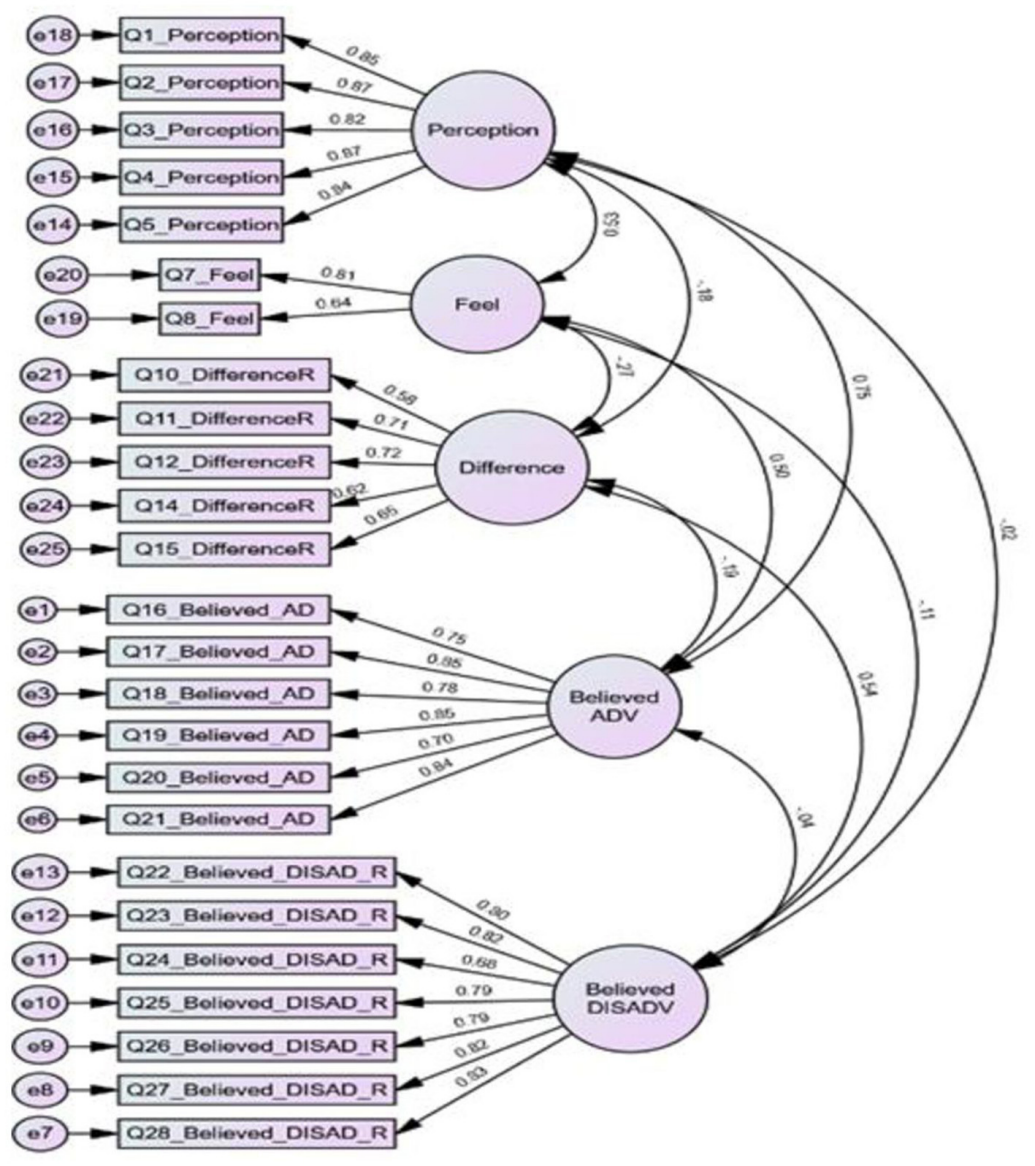
CFA model with standardized estimates values. AD, advantages; DISAD, disadvantages; R, reversed coded items; Feel, feelings.

The results of the CFA indicated a good model fit between both models, as shown in Figure 2. As per the CFA results, the observed model of the 25-item structure of the tool was verified as an excellent fit for the data [χ2_(265, N = 565)_ = 819.586, *p* < 0.001, IFI = 0.931, CFI = 0.93, RMSEA = 0.064].

Figure 2 shows the obtained *t*-values for the factor loadings ranging from 9.07 to 24.90, indicating that all items were significant at *p* < 0.001 (43). The completely standardized loadings ranged from 0.58 to 0.88.

## Discussion

Introducing new pharmacists' services is related to the high demand for and delivery of healthcare services. As drive-thru pharmacy services were established in several countries, the demand to develop a scale to assess the community perception and behaviors toward such a service became essential. This study aimed to develop and validate a tool to evaluate the public's perceptions of drive-thru community pharmacy services in Malaysia during the COVID-19 pandemic. This study is the first attempt to establish a uniform assessment tool for drive-thru community pharmacy services. The reliability and validity tests were performed. The internal consistency reliability values of the developed tool were satisfactory because of the item analysis for all the items with each of the five factors. Moreover, this study proved the validity of the developed tool using the five-factors structure: (1) believed advantages of drive-thru community pharmacy services, (2) believed disadvantages of drive-thru community pharmacy services, (3) differences between drive-thru community pharmacy services and in-store drug refill services, (4) perceptions toward drive-thru community pharmacy services, and (5) feelings regarding how the introduction of drive-thru community pharmacy services may affect the image of community pharmacists.

In this study, the 565-sample size was sufficient for the EFA, as it was more significant than the calculated sample size of 363 (10), confirming that the data were appropriate for factor analysis. The EFA was performed, identifying the factors that should have been kept in the model. An initial study was run to obtain the eigenvalues for each factor in the data. The five factors had eigenvalues of over one. By the EFA results, the structured tool achieved the five-factor structure to assess public perceptions toward drive-thru community pharmacy services. This contradicts a study by Liew et al., in which their developed tool to assess patients' satisfaction with drive-thru pharmacy services at Queen Elizabeth Hospital in Malaysia achieved 15 items for only one factor structured tool (11).

The developed tool in the current study was structured according to the recommendations by Davis (30), despite the scarcity in the literature of studies that have developed and validated assessment tools of customers' or the public's perceptions toward drive-thru community pharmacy services. Its process of development started with the identification of the tool concept by a thorough literature review, then by determining its formatting, writing, scoring, and comprehensibility, followed by the performing of validity tests by an expert panel and a factor analysis (CFA and EFA), and finally through a reliability test. This contradicts the study by Liana and Hasnah, where the team members developed a structured tool to assess patients' perceptions toward drive-thru pharmacy services in HRPZ II in Malaysia and then checked for validity by pilot tests and reliability by a Cronbach's alpha test (10).

Several previously conducted studies were different from the current study in terms of the development of the tool and the assessment design. Lee and Larson (44) developed a questionnaire to assess patients' views toward drive-thru pharmacy services for a quality service evaluation. They evaluated the developed questionnaire through face validity by the research team and additional reviewers only (44). In Taiwan, to assess the efficacy of the first drive-thru pharmacy services at Shuang-Ho Hospital, they used a pre- and post-study design method to compare the refilling system and changes in prescriptions behavior for 6 months before starting drive-thru services and 6 months after starting that service (12). Additionally, Diri structured a questionnaire to assess the need for, awareness and perception of, and barriers to drive-thru community pharmacy services during COVID-19 in Saudi Arabia, with no details provided about the validity and reliability of that developed tool (24). Finally, Abu Hammour et al. assessed Jordan's community awareness and perception of drive-thru pharmacy services with only a two-factor structured tool (23). The number of factors related to the statistical analysis (EFA and CFA) differed between the current study and Abu Hammour's study. The current study supports the five-structured factors tool theory.

### Study limitations and contributions

This study has several limitations. First, the search was limited to include only English-written journal articles. Second, the item selection process was theoretically unifying the theory of presence, and this filtering of items based on a theoretical understanding might introduce bias. The study used exploratory and confirmatory factor analyses to understand the public's perceptions of the drive-thru community pharmacy services during COVID-19. Moreover, this study highlighted the assessment aspects of these services from a public perspective. The current study is different, because this is the first research project suggesting the validity of a tool for measuring public perceptions of drive-thru pharmacy services in a community setting during COVID-19 in Malaysia. Moreover, the methodology used in this study can guide future research in developing and validating research tools for drive-thru community pharmacy services. Finally, this research may serve as a cornerstone tool for researchers to assess public perceptions about these services during COVID-19 and upcoming pandemics. It can be a reliable reference for researchers and practitioners through the five-factor structured model.

## Conclusion

As the drive-thru pharmacy service is already established in several countries, the demand for developing a scale to assess the community perception of and behavior toward such a service has become essential. The five-factor scale was successfully developed in the current study, and CFA findings indicated an acceptable fit of the five-domains model. The present study's findings are also expected to provide valuable insights to pharmacists to assess the public point of view of drive-thru community pharmacy services and the better application of such a service in different countries. Furthermore, the developed tool needs to be improved to be evaluated for use in future studies, and it needs to be explored globally for better reliability and feasibility.

## Data availability statement

The original contributions presented in the study are included in the article/Supplementary material, further inquiries can be directed to the corresponding author.

## Ethics statement

Study approval was obtained from the Human Research Ethics Committee of Universiti Sains Malaysia (USM) (Reference code: USM/JEPeM/21110755). The patients/participants provided their written informed consent to participate in this study.

## Author contributions

BA and SO: conceptualization. BA and LA: methodology, formal analysis, and data curation. BA, LA, RH, and SO: validation and writing—review and editing. BA: writing—original draft preparation and project administration. SO and RH: visualization and supervision. All authors have read and agreed to the published version of the manuscript. All authors contributed to the article and approved the submitted version.

## References

[1] Hepler CD Strand LM. Opportunities and responsibilities in pharmaceutical care. Am J Hosp Pharm. (1990) 47:533–43. 10.1093/ajhp/47.3.533 2316538

[2] Farris KB Fernandez-Llimos F Benrimoj SI. Pharmaceutical care in community pharmacies: practice and research from around the world. Ann Pharmacother. (2005) 39:1539–41. 10.1345/aph.1G049 16014373

[3] Moullin JC Sabater-Hernández D Fernandez-Llimos F Benrimoj SI. Defining professional pharmacy services in community pharmacy. Res Soc Adm Pharm. (2013) 9:989–95. 10.1016/j.sapharm.2013.02.005 23591411

[4] Berbatis CG Sunderland VB Joyce A Bulsara M Mills C. Enhanced pharmacy services, barriers and facilitators in Australia's community pharmacies: Australia's National Pharmacy Database Project. Int J Pharm Pract. (2010) 15:185–91. 10.1211/ijpp.15.3.0005

[5] Loh P Chua SS Karuppannan M. The extent and barriers in providing pharmaceutical care services by community pharmacists in Malaysia: a cross-sectional study. BMC Health Serv Res. (2021) 21:822. 10.1186/s12913-021-06820-7 34399749 PMC8365940

[6] Myers A. Drive-Through Businesses. American Business History and Civil Liberties (2011).

[7] Drive Thru Pharmacy Griffith Soul Pattinson Chemist. Drive Thru Pharmacy Griffith (2010). Available from: https://www.drivethrupharmacy.com.au/ (accessed October 29, 2022).

[8] First Drive-Through Pharmacy Opens in Zagreb,. The Dubrovnik Times (2017). Available from: https://www.thedubrovniktimes.com/news/croatia/item/1879-first-drive-through-pharmacy-opens-in-zagreb (accessed October 30, 2022).

[9] Abu Farha R Abu Hammour K Alefishat E Alsaeed H Alma'aiah S. Drive-thru pharmacy service: assessments of awareness, perception and barriers among pharmacists in Jordan. Saudi Pharm J. (2017) 25:1231–6. 10.1016/j.jsps.2017.09.008 29204073 PMC5688229

[10] Liana AN Hasnah I. Drive-thru pharmacy service: assessment of perception among patients or caregivers in Hospital Raja Perempuan Zainab II. Int J Pharm Pharm Sci. (2015) 7:212–5.29204073

[11] Liew JES Abdul Gapar AA Bin Shim LT. Evaluation of drive-through pharmacy service in Queen Elizabeth Hospital Malaysia. J Pharm Policy Pract. (2020) 13:1–8. 10.1186/s40545-020-00221-7 32537169 PMC7288545

[12] Lin YF Lin YM Sheng LH Chien HY Chang TJ Zheng CM. First drive-through pharmacy services in Taiwan. J Chin Med Assoc. (2013) 76:37–41. 10.1016/j.jcma.2012.10.001 23331780

[13] Boots Opens Britain's First Drive-Through Chemist,... in an Old McDonald's. Daily Mail Online (2008). Available from: https://www.dailymail.co.uk/health/article-1047196/Boots-opens-Britains-drive-chemist–old-McDonalds.html (accessed October 29, 2022).

[14] Jacqueline A Padilla Erwin M. Faller Drive-Thru community pharmacy in the “New Normal Era”: an innovation in pharmaceutical services and its socio-economic impact GSC. Biol Pharm Sci. (2022) 18:137–54. 10.30574/gscbps.2022.18.3.0101

[15] Hussain R Dawoud DM Babar ZUD. Drive-thru pharmacy services: a way forward to combat COVID-19 pandemic. Res Social Adm Pharm. (2021) 17:1920–4. 10.1016/j.sapharm.2020.07.015 32792322 PMC7373674

[16] Ababneh BF Ong SC Mahmoud F Alsaloumi L Hussain R. Attitudes, awareness, and perceptions of general public and pharmacists toward the extended community pharmacy services and drive-thru pharmacy services: a systematic review. J Pharm Policy Pract. (2023) 16:37. 10.1186/s40545-023-00525-4 36864499 PMC9979876

[17] Loh BC Wah KF Teo CA Khairuddin NM Fairuz FB Liew JE. Impact of value added services on patient waiting time at the ambulatory pharmacy Queen Elizabeth Hospital. Pharm Pract. (2017) 15:846. 10.18549/PharmPract.2017.01.846 28503218 PMC5386619

[18] Farmasi komuniti pandu lalu pertama di Malaysia [METROTV]. (2022). Available from: https://www.hmetro.com.my/mutakhir/2022/02/806771/farmasi-komuniti-pandu-lalu-pertama-di-malaysia-metrotv (accessed September 24, 2022).

[19] Christensen DB Penna PM. Quality assessment and quality assurance of pharmacy services. J Manag Care Pharm. (2015) 2:40–51. 10.18553/jmcp.1995.1.1.40

[20] Prakash B. Patient satisfaction. J Cutan Aesthet Surg. (2010) 3:151. 10.4103/0974-2077.74491 21430827 PMC3047732

[21] Boudreaux ED O'Hea EL. Patient satisfaction in the emergency department: a review of the literature and implications for practice. J Emerg Med. (2004) 26:13–26. 10.1016/j.jemermed.2003.04.003 14751474

[22] Saiboon I Siew Eng H Krishnan B Nooraini Ali S Murad N Pathnathan A. A study of patients' satisfaction with the emergency department (ED) of Hospital Universiti Kebangsaan Malaysia (HUKM). Med Heal. (2008) 3:7–13.

[23] Abu Hammour K Abu Farha R Rizik M Mukattash T Alnan M Alkhader A. Pharmacy drive-thru service in Jordan: assessing customers' awareness, perceptions and factors affecting the use of this service. J Pharm Heal Serv Res. (2019) 10:141–7. 10.1111/jphs.12245

[24] Diri RM. The impact of COVID-19 outbreak on reassessing the need for drive thru community pharmacy: cross-sectional study. J Microsc Ultrastruct. (2020) 8:162–4. 10.4103/JMAU.JMAU_65_20 33623741 PMC7883499

[25] Chui MA Halton K Peng JM. Exploring patient-pharmacist interaction differences between the drive-through and walk-in windows. J Am Pharm Assoc. (2009) 49:427–31. 10.1331/JAPhA.2009.07165 19443324

[26] Sample Size Calculator by Raosoft Inc. Available from: http://www.raosoft.com/samplesize.html (accessed January 5, 2023).

[27] Davis AE. Instrument development: getting started. J Neurosci Nurs. (1996) 28:204–7. 10.1097/01376517-199606000-00009 8818987

[28] Bolarinwa O. Principles and methods of validity and reliability testing of questionnaires used in social and health science researches. Niger Postgrad Med J. (2015) 22:195–201. 10.4103/1117-1936.173959 26776330

[29] Hassan ZA Schattner P Mazza D. Doing a pilot study: why is it essential? Malays Fam Phys. (2006) 1:70–3. 27570591 PMC4453116

[30] Cronbach LJ. Coefficient alpha and the internal structure of tests. Psychometrika. (1951) 16:297–334. 10.1007/BF02310555

[31] Fabrigar LR MacCallum RC Wegener DT Strahan EJ. Evaluating the use of exploratory factor analysis in psychological research. Psychol Methods. (1999) 4:272–99. 10.1037/1082-989X.4.3.27219609833

[32] Taherdoos H Sahibuddin S Jalaliyoon N. Exploratory factor analysis; Concepts and theory. Adv Appl Pure Math. (2022) 27:375–82. Available online at: https://hal.archives-ouvertes.fr/hal-02557344/document

[33] Wood ND Akloubou Gnonhosou DC Bowling J. Combining parallel and exploratory factor analysis in identifying relationship scales in secondary data. Marriage Fam Rev. (2015) 51:385–95. 10.1080/01494929.2015.1059785 26494935 PMC4610406

[34] Hooper D Coughlan J Mullen MR. Structural equation modelling: guidelines for determining model fit. Electron J Bus Res Methods. (2008) 6:53–60. 10.21427/D7CF7R

[35] Hu L Bentler PM. Cutoff criteria for fit indexes in covariance structure analysis: conventional criteria versus new alternatives. Struct Equ Model. (1999) 6:1–55. 10.1080/1070551990954011836787513

[36] Kline RB. Principles and Practice of Structural Equation Modeling. 3rd ed. New York, NY: Guilford Press (2011). xvi, 427–xvi, 427. (Methodology in the Social Sciences.).

[37] Brown TA. Confirmatory Factor Analysis for Applied Research. 2nd ed. New York, NY; London: Guilford Press (2015).

[38] Maat SM Adnan M Abdullah MFNL Ahmad CNC Puteh M. Confirmatory factor analysis of learning environment instrument among high performance school students. Creat Educ. (2015) 06:640–6. 10.4236/ce.2015.66063

[39] Browne MW Cudeck R. Alternative ways of assessing model fit. Sociol Methods Res. (1992) 21:230–58. 10.1177/0049124192021002005

[40] Kaiser HF. An index of factorial simplicity. Psychometrika. (1974) 39:31–6. 10.1007/BF02291575

[41] Horn JL. A rationale and test for the number of factors in factor analysis. Psychometrika. (1965) 30:179–85. 10.1007/BF02289447 14306381

[42] Maskey R Fei J Nguyen HO. Use of exploratory factor analysis in maritime research. Asian J Shipp Logist. (2018) 34:91–111. 10.1016/j.ajsl.2018.06.006

[43] Hatcher L. A Step-by-Step Approach to Using the SAS System for Factor Analysis Structural Equation Modeling. (1994). 588 p. Available from: https://books.google.com/books/about/A_Step_by_Step_Approach_to_Using_the_SAS.html?id=Liz6EWrUuXEC (accessd December 7, 2022).

[44] Lee TA Larson LN. Evaluating the use and quality of pharmacy drive-up services. J Am Pharm Assoc. (1999) 39:338–45. 10.1016/S1086-5802(16)30447-8 10363460

