# Supplementary material for: Development and validation of an assessment tool for public perceptions toward drive-thru community pharmacy services in Malaysia during COVID-19
Source: Front Public Health. 2023 Aug 3;11:1144466. doi: 10.3389/fpubh.2023.1144466 (PMC10434766; doi:10.3389/fpubh.2023.1144466)
Supplement: Supplementary file 1 [file Table_1.DOCX]

**Appendix 1**

**Factor loadings by EFA (Pattern Matrix of the factors and item).**

| **Item code** | **Factors** | | | | |
| --- | --- | --- | --- | --- | --- |
|  | 1 | 2 | 3 | 4 | 5 |
| Q20_Believed_AD | 0.856 |  |  |  |  |
| Q19_Believed_AD | 0.818 |  |  |  |  |
| Q21_Believed_AD | 0.807 |  |  |  |  |
| Q18_Believed_AD | 0.790 |  |  |  |  |
| Q17_Believed_AD | 0.746 |  |  |  |  |
| Q16_Believed_AD | 0.661 |  |  |  |  |
| Q28_Believed_DISAD_R |  | 0.860 |  |  |  |
| Q27_Believed_DISAD_R |  | 0.856 |  |  |  |
| Q23_Believed_DISAD_R |  | 0.850 |  |  |  |
| Q24_Believed_DISAD_R |  | 0.808 |  |  |  |
| Q22_Believed_DISAD_R |  | 0.799 |  |  |  |
| Q25_Believed_DISAD_R |  | 0.767 |  |  |  |
| Q26_Believed_DISAD_R |  | 0.747 |  |  |  |
| Q11_DifferenceR |  |  | 0.769 |  |  |
| Q10_DifferenceR |  |  | 0.704 |  |  |
| Q12_DifferenceR |  |  | 0.702 |  |  |
| Q15_DifferenceR |  |  | 0.682 |  |  |
| Q14_DifferenceR |  |  | 0.639 |  |  |
| Q13_Difference | 0.341* | 0.069 | -0.352 | 0.211 | -0.122 |
| Q1_Perception |  |  |  | 0.952 |  |
| Q5_Perception |  |  |  | 0.862 |  |
| Q3_Perception |  |  |  | 0.847 |  |
| Q4_Perception |  |  |  | 0.832 |  |
| Q2_Perception |  |  |  | 0.797 |  |
| Q9_Difference | 0.232 | -0.100 | -0.173 | 0.343* | 0.152 |
| Q6_FeelR | 0.170 | 0.047 | 0.454* | 0.090 | -0.395 |
| Q8_Feel |  |  |  |  | 0.795 |
| Q7_Feel |  |  |  |  | 0.663 |

*Cross-loading items and excluded from the CFA. Extraction method: Principal component analysis. Rotation Method: Oblimin with Kaiser Normalization. Rotation converged in 8 iterations. Q: Question. AD: Advantages. DISAD: Disadvantages. R: Reversed coded items. Feel: Feelings.
