# Supplementary material for: Development and validation of an assessment tool for public perceptions toward drive-thru community pharmacy services in Malaysia during COVID-19
Source: Front Public Health. 2023 Aug 3;11:1144466. doi: 10.3389/fpubh.2023.1144466 (PMC10434766; doi:10.3389/fpubh.2023.1144466)
Supplement: Supplementary file 2 [file Table_2.DOCX]

**Appendix 2. Final developed tool to assess perceptions of the public regarding drive-thru community pharmacy service in Malaysia during COVID-19.**

**First part:** Demographics.

| **Age (Years) write your age** |  | | | | | |
| --- | --- | --- | --- | --- | --- | --- |
| **Gender** | Male | Female |  |  |  |  |
| **Marital Status** | Single | Married | Divorced | Widowed |  |  |
| **Having children** | Yes | No |  |  |  |  |
| **Area of residency** | Kuala Lumpur | Penang | Malacca | Kedah | Kelantan | Negeri Sembilan |
|  | Johor | Pahang | Perak | Perlis | Sabah | Sarawak |
|  | Selangor | Terengganu | Labuan | Putrajaya |  |  |
| **Educational level** | No formal education | Primary School | High School | Diploma | Pre-University | Bachelor’s degree |
|  | Master’s or Ph.D. degree |  |  |  |  |  |
| **Employment status** | Employed | Non-employed | Retired |  |  |  |
| **Health professional** | Yes | No |  |  |  |  |
| **Student** | Yes | No |  |  |  |  |

**Second part:** Attitudes towards community pharmacy drive-thru service.

| **Attitudes towards drive-thru community pharmacy service** |
| --- |
| **The number of community pharmacies visited last month** |
| 1. None |
| 2. One pharmacy |
| 3. Two pharmacies |
| 4. Three or more pharmacies |
| **Reasons to visit the community pharmacy (more than 1 option could be chosen)** |
| 1. Over the counter medications |
| 2. For beauty products |
| 3. Prescribed medications |
| 4. Medical Device |
| 5. Medical consultation |
| 6. Kid supply |
| 7. COVID-19 prevention supplies such as masks and hygiene products. |
| 8. Others (………………………….…) |
| **Which category will benefit the most from drive-thru community pharmacy service?** |
| 1. All population |
| 2. Women |
| 3. Geriatrics |
| 4. People with disabilities |
| **Presence of drive-thru community pharmacy at your city** |
| 1. Yes |
| 2. No |
| 3. Don’t know |
| **If yes, have you tried drive-thru community pharmacy service?** |
| 1. Yes |
| 2. No |
| 3. Not applicable |
| **If yes, how do you evaluate your experience with drive-thru community pharmacy service?** |
| 1. Excellent |
| 2. Good |
| 3. Fair |
| 4. Poor |
| 5. Not applicable |
| **If you are going to request an order at a community pharmacy using drive-thru service, what is your preferred method to do that order?** |
| 1- Through a drive-thru window |
| 2- Through WhatsApp |
| 3- Over the phone |
| 4- Online through application |
| 5- Through email |
| **If you are going to use a drive-thru service at a community pharmacy, what is your preferred method to get information about your medications(counselling)?** **(more than 1 option could be chosen)** |
| 1- Briefly through the drive-thru window |
| 2- Printed brochure given with the order |
| 3- Written on WhatsApp |
| 4- Verbally over the phone |
| 5- Through a personal visit |
| 6- Through email |
| **Where did you get information regarding drive-thru community pharmacy(more than 1 option could be chosen)** |
| 1. Pharmacy staff |
| 2. Doctors |
| 3. Leaflets |
| 4. Television |
| 5. Internet |
| 6. Friends or Colleagues |
| 7. Don’t know |
| **Are you supportive to establish drive-thru service at community pharmacies?** |
| 1. Yes |
| 2. No |

**Third part:** Perceptions towards community pharmacy drive-thru service.

| **Variables** | **Strongly agree** | **Agree** | **Neutral** | **Disagree** | **Strongly disagree** |
| --- | --- | --- | --- | --- | --- |
| **Perceptions towards drive-thru community pharmacy service as an impact of COVID-19 or even later on** |  |  |  |  |  |
| 1. I believe the introduction of drive-thru service makes the community pharmacy services more efficient. |  |  |  |  |  |
| 2. I believe that drive-thru community pharmacy service is a friendly service provided by the pharmacy during COVID-19 time or even later on. |  |  |  |  |  |
| 3. I believe that drive-thru community pharmacy service may improve my satisfaction with the pharmacy profession. |  |  |  |  |  |
| 4. I am supportive of the introduction of drive-thru service to community pharmacy practice during COVID-19 time. |  |  |  |  |  |
| 5. I am supportive to create community pharmacies with drive-thru services all over Malaysia. |  |  |  |  |  |
| **How do you feel the image of the community pharmacists will be affected by the introduction of drive-thru service?** |  |  |  |  |  |
| 1. Community pharmacists will have a good balance between the health of patients and the business side of their work. |  |  |  |  |  |
| 2. Community pharmacists will appear more concerned with the health of patients than with the business side of their work. |  |  |  |  |  |
| **Differences between the drive-thru community pharmacy service and in-store drug refill services.** |  |  |  |  |  |
| 1. Pharmacists might be less available to answer questions using drive-thru service compared to in-store refill. |  |  |  |  |  |
| 2. Written information might be less supplied using drive-thru pharmacy service compared to in-store refill. |  |  |  |  |  |
| 3. Pharmacists cannot explain important points about prescriptions while providing drive-thru service compared to that in-store refill. |  |  |  |  |  |
| 4. Unlike in-store service, drive-thru service is suitable only for refill prescriptions but not for new prescriptions. |  |  |  |  |  |
| 5. Unlike in-store service, drive-thru service is suitable only for OTC but not for prescriptions medications. |  |  |  |  |  |
| **Believed advantages towards the drive-thru community pharmacy service as an impact of COVID-19** |  |  |  |  |  |
| 1. Drive-thru community pharmacy service may help me get my medications on time without delay. |  |  |  |  |  |
| 2. Drive-thru community pharmacy will be helpful during COVID-19 time and quarantine time. |  |  |  |  |  |
| 3. Drive-thru community pharmacy service has the advantage of serving sick patients, elderly, or disabled people during COVID-19 time. |  |  |  |  |  |
| 4. Drive-thru pharmacy service enhances social distancing and reduces the spread of the COVID-19 virus. |  |  |  |  |  |
| 5. Drive-thru community pharmacy service reduces the pressure on health care centers during COVID-19 time. |  |  |  |  |  |
| 6. Drive-thru community pharmacy service is needed to be implemented in most community pharmacies during COVID-19 time or even later on for getting medications or supplies. |  |  |  |  |  |
| **Believed disadvantages towards the drive-thru community pharmacy service** |  |  |  |  |  |
| 1. Drive-thru community pharmacy service may contribute to dispensing errors due to the fast service provided. |  |  |  |  |  |
| 2. Drive-thru community pharmacy service may contribute to communication errors between the patient and pharmacist. |  |  |  |  |  |
| 3. Drive-thru community pharmacy service may need extra money to offer drive-thru windows. |  |  |  |  |  |
| 4. Drive-thru community pharmacy service is not convenient in providing drug information/counselling to patients (especially written information). |  |  |  |  |  |
| 5. Getting prescriptions dispensed as quickly as possible using drive-thru community pharmacy service, the quality of pharmacy service will drop. |  |  |  |  |  |
| 6. Drive-thru community pharmacy service restricts the opportunity for interaction with the pharmacist because the customer feels they can't ask questions while they're being hurried through. |  |  |  |  |  |
| 7. Drive-thru community pharmacy service restricts the opportunity for interaction with the pharmacist because the pharmacist will not be able to offer any level of interaction. |  |  |  |  |  |
